# Effect of degree of hydrolysis of whey protein on in vivo plasma amino acid appearance in humans

**DOI:** 10.1186/s40064-016-1995-x

**Published:** 2016-03-31

**Authors:** Jean Farup, Stine Klejs Rahbek, Adam C. Storm, Søren Klitgaard, Henry Jørgensen, Bo M. Bibby, Anja Serena, Kristian Vissing

**Affiliations:** Section for Sport Science, Department of Public Health, Aarhus University, Dalgas Avenue 4, 8000 Aarhus, Denmark; Department of Animal Science, Aarhus University, Aarhus, Denmark; Department of Biostatistics, Aarhus University, Aarhus, Denmark; Arla Foods Ingredients Group P/S, Aarhus, Denmark

**Keywords:** Whey protein hydrolysate, Degree of hydrolysis, Plasma appearance

## Abstract

Whey protein is generally found to be faster digested and to promote faster and higher increases in plasma amino acid concentrations during the immediate ~60 min following protein ingestion compared to casein. The aim of the present study was to compare three different whey protein hydrolysates with varying degrees of hydrolysis (DH, % cleaved peptide bonds) to evaluate if the degree of whey protein hydrolysis influences the rate of amino acid plasma appearance in humans. A casein protein was included as reference. The three differentially hydrolysed whey proteins investigated were: High degree of hydrolysis (DH, DH = 48 %), Medium DH (DH = 27 %), and Low DH (DH = 23 %). The casein protein was intact. Additionally, since manufacturing of protein products may render some amino acids unavailable for utilisation in the body the digestibility and the biological value of all four protein fractions were evaluated in a rat study. A two-compartment model for the description of the postprandial plasma amino acid kinetics was applied to investigate the rate of postprandial total amino acid plasma appearance of the four protein products. The plasma amino acid appearance rates of the three whey protein hydrolysates (WPH) were all significantly higher than for the casein protein, however, the degree of hydrolysis of the WPH products did not influence plasma total amino acid appearance rate (estimates of DH and 95 % confidence intervals [CI] (mol L^−1^ min^−1^): High DH 0.0585 [0.0454, 0.0754], Medium DH 0.0594 [0.0495, 0.0768], Low DH 0.0560 [0.0429, 0.0732], Casein 0.0194 [0.0129, 0.0291]). The four protein products were all highly digestible, while the biological value decreased with increasing degree of hydrolysis. In conclusion, the current study does not provide evidence that the degree of whey protein hydrolysis is a strong determinant for plasma amino acid appearance rate within the studied range of hydrolysis and protein dose.

**Electronic supplementary material:**

The online version of this article (doi:10.1186/s40064-016-1995-x) contains supplementary material, which is available to authorized users.

## Background

Maintenance of skeletal muscle mass and function throughout life is essential for mobility, healthy living and quality of life. Ingestion of dietary protein is necessary for maintaining skeletal muscle mass. Furthermore, the quality (i.e. the essential amino acid profile), the amount of protein ingested, protein absorption kinetics, and protein digestibility all constitute crucial factors for the postprandial stimulation of muscle protein synthesis (Cuthbertson et al. 2005; Volpi et al. 2003).

The essential amino acids (EAAs) in a protein supplement are primarily responsible for stimulation of MPS following protein ingestion (Tipton et al. 1999b; Volpi et al. 2003). In this regard, the amino acid induced stimulation of MPS in the rested state has been found to increase in a dose-dependent manner until a threshold of approximately 10 g of EAA is reached, whereas no further increase in MPS is observed with 20 or 40 g of EAA (Cuthbertson et al. 2005). The essential amino acid, leucine, has received much attention, since animal studies has reported leucine to be a potent activator of anabolic signalling pathways involving the mammalian target of rapamycin (mTOR). Cell culture studies have demonstrated that leucine is able to induce protein synthesis via interaction with mTOR (Dennis et al. 2011; Proud 2014) and animal studies have reported that leucine alone is sufficient to stimulate mTOR signalling (Crozier et al. 2005; Suryawan et al. 2008). In human in vivo studies leucine supplementation constitutes a potent means to stimulate MPS (Churchward-Venne et al. 2012, 2014). Additionally, absence of leucine in an EAA supplement, has been observed to reduce activation of mTOR signalling which potentially could reduce the MPS (Moberg et al. 2014). The latter observation has given rise to the suggestion that leucine may be a MPS “trigger” so that protein supplements rich in leucine would be more effective at stimulating MPS than supplements with a lower leucine content (Phillips 2014).

In addition to the leucine content, it has been suggested that the rapidity of protein digestion, and thus the peak plasma concentration of leucine may be an important factor for achieving maximal MPS (Phillips 2014). Thus, the protein absorption kinetics may also be important factor. Slowly absorbed proteins, such as casein that precipitates with acid, elicits a low but sustained increase in MPS and a decrease in protein breakdown, while fast absorbed protein, such as whey protein that remains soluble in the stomach, causes high and short increases in MPS (Boirie et al. 1997). As such, whey protein is considered to be superior (Reitelseder et al. 2011) to casein in supporting MPS during the immediate 4 h following ingestion (Burd et al. 2012; Tang et al. 2009). This may relate to the higher plasma concentrations of EAA and leucine during the immediate ~60 min following ingestion of whey compared to casein (Burd et al. 2012; Reitelseder et al. 2011). It is important to note, that when whey protein is ingested in small doses (“pulse feeding”), to mimic the absorption kinetics of casein protein, the effect on MPS is also reduced (West et al. 2011). This may indicate that the superiority of whey protein is not solely related to the greater leucine content in whey compared to casein (Phillips 2014) and that the faster absorption kinetics of whey protein are important for MPS as well. The absorption rate of both whey and casein protein can be increased by protein hydrolysis (measured as degree of protein hydrolysis, i.e., % cleaved peptide bonds) (Morifuji et al. 2010; Power et al. 2009), which may potentially influence MPS.

Regarding protein digestibility, the process of manufacturing protein solutions may render some amino acids unavailable, due to e.g. heat-treatment, so that they cannot be utilized by the body (Rutherfurd and Moughan 2012). Amino acids, especially the sulphur-containing amino acids, may be susceptible to oxidation, with subsequent loss of bioavailability (Rutherfurd and Moughan 2012). Therefore, digestibility could be an important aspect to consider when comparing proteins that are susceptible to damage from processing. Protein quality has traditionally been evaluated by the protein digestibility-corrected amino acid score (PDCAAS). This estimate of quality is derived from measures of the limiting EAA content in the protein, compared with the preschool-age child amino acid requirement, multiplied by the digestibility (measured in a rat assay) of the analysed protein (Schaafsma 2005).

The aim of this study was to investigate if different degrees of whey protein hydrolysis would result in differentiated plasma total amino acid appearance rates in protein products high in EAAs. To pursue this aim, we compared three different whey protein hydrolysate products with different degrees of hydrolysis. An intact casein protein was included as reference. Comparisons were made on the following parameters; (1) the rate of plasma appearance of total amino acids in humans, and; (2) the digestibility of all four protein products (assessed in a rat study).

We hypothesised; (1) that the rate of plasma appearance of total amino acids (TAA) would increase according to degree of hydrolysis of the whey protein, i.e., the higher degree of hydrolysis the faster the plasma appearance of TAA and (2) that the digestibility of the four protein products would be equal.

## Methods

### Test protein profiles

**Fig. 1:**
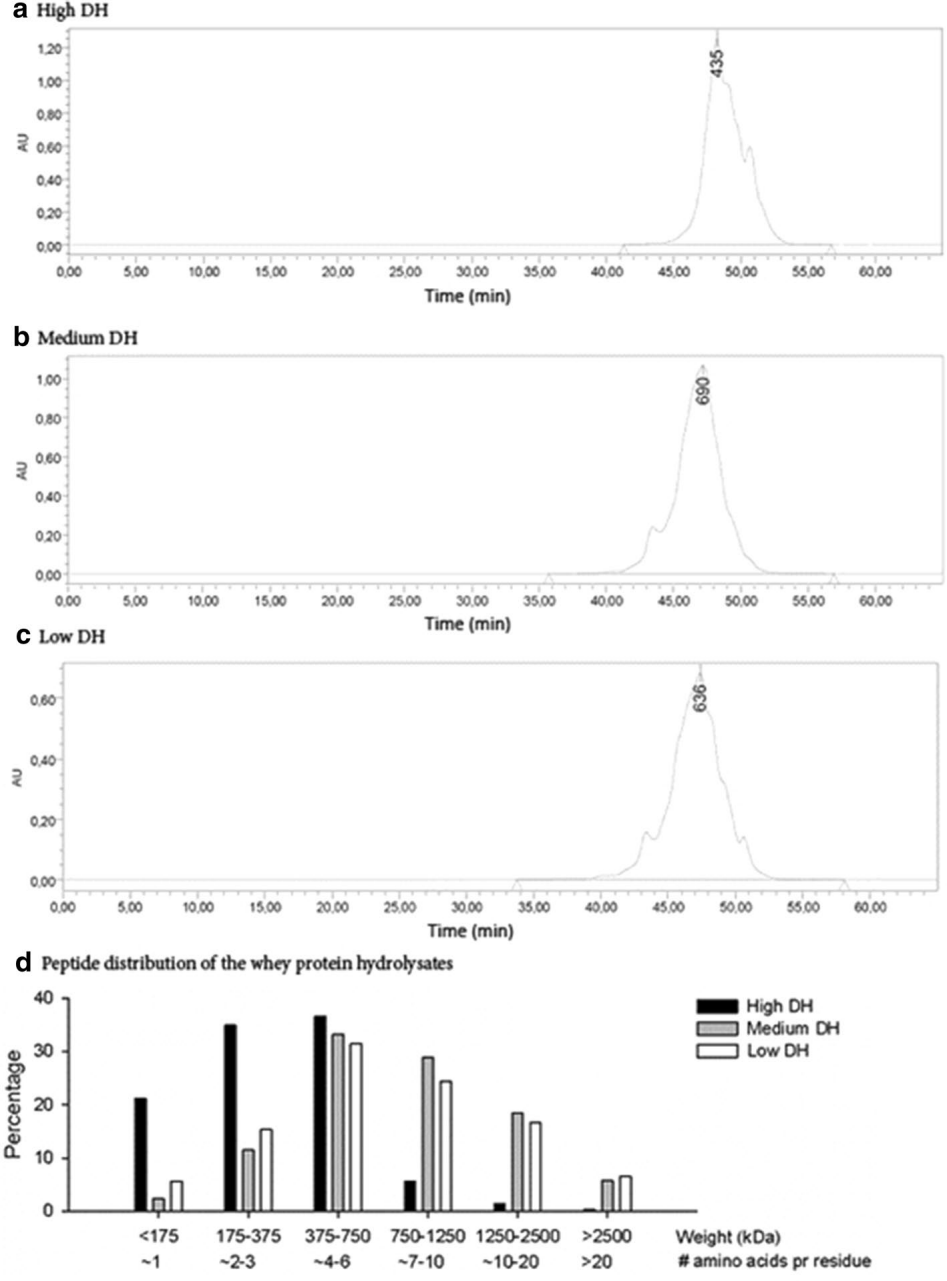
Chromatograms and peptide distributions of the three whey protein hydrolysates. DH, Degree of Hydrolysis (% cleaved peptide bonds). AU, Arbitrary Units. **a**–**c** Chromatograms showing molecular weight distribution of the High DH (**a**), Medium DH (**b**), and Low DH (**c**) supplements. Peak molecular weight is denoted for each supplement. **d** The peptide distribution of the three WPH supplements was generated from the chromatograms. The weight category <175 kDa is an estimate of free amino acids, 175–375 kDa is an estimate for di- and tri-peptides, 375–750 kDa is an estimate for peptides containing 4–6 amino acids, 750–1250 kDa is an estimate for peptides containing 7–10 amino acids, 1250–2500 kDa is an estimate for peptides containing 10–20 amino acids, >2500 kDa is an estimate for proteins containing more than 20 amino acids

Three WPH fractions with different degrees of hydrolysis and an intact casein protein included as reference were produced by Arla Foods Ingredients Group for this study. Specifications of the four protein products are listed in Table 1. The nitrogen content was measured by the Dumas procedure (Hansen 1989) and protein was calculated as nitrogen × 6.38, lactose content was measured by the galacto-oligosaccharide method (Bertelsen and Langborg 2012), fat content was measured by a gravimetric reference method [International Dairy Federation—Milk Determination of Fat-Content—Gravimetric Method (Reference Method)—Provisional International Idf Standard Ib 1983 (1983)], and degree of protein hydrolysis was measured by the *o*-phthaldialdehyde (OPA) method (Nielsen et al. 2001). The analysis of the amino acid profiles were performed in accordance with the EU regulation concerning the methods of sampling and analysis for the official control of feed (Regulation 2009). The molecular weight distribution was analysed using gel filtration chromatography on 3 serial 7.8 × 300 mm columns loaded with TSK G2000SWXL gel (Tosoh Bioscience LLC, Japan). The mobile phase was a 0.04 M phosphate buffer with 0.4 M ammonium chloride, 0.1 % trifluoroacetic acid, and 25 % acetonitrile, with a flow rate of 0.7 mL/min. Chromatograms of the molecular weight distribution of the three WPH are shown in Fig. 1a–c. The peptide distribution (Fig. 1d) of the three WPH supplements was generated from the chromatograms.

**Table 1:** Characterization of the High DH, Medium DH, Low DH, and Casein protein fractions

	High DH	Medium DH	Low DH	Casein
Protein, as is (%), (N × 6.38)	77	76	80	72
Lactose, as is (%)	2.7	2.5	3.7	0.2
Fat, as is (%)	0.1	0.1	6.4	1.3
Degree of hydrolysis (% cleaved peptide bonds)	48	27	23	NA
*Amino acid profiles* (*AA/total protein*, *%*)				
Leucine	16.2	8.0	9.8	9.2
Isoleucine	6.1	5.3	5.5	4.8
Valine	7.2	4.8	5.2	6.3
Lysine	7.1	9.7	8.8	7.8
Methionine	1.9	1.7	2.0	2.7
Phenylalanine	5.4	2.2	3.0	4.9
Threonine	6.9	7.1	6.7	4.0
Tryptophane	2.5	1.0	1.6	1.2
Alanine	7.4	4.5	4.8	3.0
Arginine	2.5	2.1	2.2	3.3
Aspartic acid	6.5	11.3	10.4	6.9
Cysteine	0.9	2.2	2.3	0.5
Glutamic acid	9.4	18.8	16.7	21.1
Glycine	2.5	1.7	1.9	1.8
Histidine	2.3	1.7	1.7	2.8
Proline	1.7	6.5	5.9	10.5
Serine	6.7	4.5	4.9	5.4
Tyrosine	6.9	7.1	6.7	4.0
Σ essential amino acids	53.3	39.7	42.5	40.7

*N* nitrogen. *DH* degree of hydrolysis, *NA* not analyzed. *AA* amino acids

### Protein digestibility study with rats (PDCAAS)

The care and housing of animals used in this study were in compliance with Danish laws and regulations for the humane care and use of animals in research (The Danish Ministry of Justice, Animal Testing Act, Consolidation Act no 1306 of November 23, 2007 and performed under licence obtained from the Danish Animal Experimentation Inspectorate, Ministry of Food, Agriculture and Fisheries). The experiments were carried out with four groups of five male Wistar rats approximately 3 weeks old, weighing (mean ± SEM) 97.45 ± 0.91 g, obtained from Taconic Europa, Lille Skensved, Denmark. The rats were randomly assigned to one of the four protein diet groups. The rats were adapted to their diets for 4 days followed by a balance period of 5 days, during which faeces and urine were collected. Fresh water was available and 10 g dry matter feed was supplied every day. The rats were housed individually in plexiglas cages with stainless-steel mesh floors, which permitted separate collection of urine and feces. The cages were kept in a single room with controlled temperature (25 °C), relative humidity at 60 %, and 12-h light and dark cycles. The four diets (Table 2) containing the different protein sources were adjusted to 150 mg N/kg dry matter (DM) with the appropriate proportion of a N-free mixture consisting of 80.7 % autoclaved maize starch (Cerestar Scandinavia, Charlottenlund, Denmark), 8.9 % sucrose (Danisco Sugar, Copenhagen, Denmark), 5.2 % cellulose (MN grade 100, Macherey–Nagel GmbH, Düren, Germany), and 5.2 % soy bean oil (AarhusKarlsham Denmark, A/S, Aarhus, Denmark). The diets were supplemented with the necessary amounts of minerals and vitamins according to National Research Council (NRC) recommendations for growing rats (1995). Throughout the adaptation and balance periods, each animal received 10 g of DM and 150 mg of N per day. At the end of the balance period the rats were weighed and food intake was determined.

**Table 2:** Ingredient composition for the experimental diet as fed to the rats

	High DH	Medium DH	Low DH	Casein
Protein source (%)	11.0	11.6	10.2	12.4
N-free mixture (%)*	84.5	83.9	85.4	83.1
Mineral/vitamin mix (%)^†,‡^	4.5	4.5	4.5	4.5

*DH* degree of hydrolysis (% cleaved peptide bonds)

* Composition of N-free mixture (g/kg): Maize starch, autoclaved 807 g; sucrose, 89 g; cellulose powder, 52 g; soya bean oil, 52 g

^†^The mineral mixture supplies to the rats (per kg diet): 1.2 g Ca as CaCO_3_; 2.5 g Ca as Ca_3_(C_6_H_5_O_7_)_2_; 1.4 g Ca and 1.1 g P as CaHPO_4_ × 2H_2_O; 2.1 g K and 1.7 g P as KH_2_PO_4_; 2.4 g K as KCl; 0.6 g Na as NaCl; 0.3 g Mg as MgSO_4_; 0.3 g Mg as 4MgCO_3_ × Mg(OH)_2_ × 5H_2_O; 8 mg Cu as CuSO_4_ × 5H_2_O; 47 mg Fe as Ammonium ferric citrate; 12 mg Mn as MnSO_4_ × H_2_O; 18 mg Zn as ZnSO_4_ × 7H_2_O; 143 µg I as KI; 146 µg Se as Na_2_SeO_3_ × 5H_2_O

^‡^The vitamin mixture supplies to the rats (per kg diet): retinol acetate, 0.7 mg; cholecalciferol, 0.025 mg; all-rac-a-tocopherol acetate, 28 mg; menadione sodium bisulfite, 1.0 mg; biotin, 0.2 mg; choline chloride, 1013 mg; folic acid, 1.0 mg; nicotinamide, 15.2 mg; Ca-pantothenate, 10.2 mg; riboflavin, 3.2 mg; thiamine × HCl, 4.1 mg; pyridoxine × HCl, 6.1 mg; cyanocobalamin, 51 mg

### Chemical analysis and calculation of biological value, true digestibility and PDCAAS

The nitrogen content of the diet intake (N_intake_), the faeces (N_faeces_) and the urine (N_urine_) were measured by the Dumas procedure (Hansen 1989) and protein was calculated as N × 6.38. The nitrogen metabolized (N_metabolic_) during the balance period has previously been estimated to 1.01 mg N/g drymatter feedstuff (Jørgensen et al. 1997). The endogenous nitrogen (N_endogenous_) i.e. nitrogen excreted that does not originate from the test diet, was estimated to 15.2 mg/day for a growing rat (Eggum 1973). True digestibility (TD) and biological value (BV) were calculated using the following equations:

1 $${\text{TD}} = \frac{{{\text{N}}_{\text{intake}} - \left( {{\text{N}}_{\text{faeces}} - {\text{N}}_{\text{metabolic}} } \right)}}{{{\text{N}}_{\text{intake}} }}$$

2 $${\text{BV}} = \frac{{{\text{N}}_{\text{intake}} - \left( {{\text{N}}_{\text{faeces}} - {\text{N}}_{\text{metabolic}} } \right) - \left( {{\text{N}}_{\text{urine}} - {\text{N}}_{\text{endogenous}} } \right) }}{{{\text{N}}_{\text{intake}} - \left( {{\text{N}}_{\text{faeces}} - {\text{N}}_{\text{metabolic}} } \right)}}$$

The PDCAAS value was calculated as the amount of the first limiting indispensable amino acid in the test protein, as a fraction of the amount of the corresponding amino acid recommended in an age specific reference pattern, multiplied with the true faecal digestibility, as measured in the rat assay. The essential amino acid requirement stated by FAO/WHO 2007/2011 of 3–10 year old children was used for the reference amino acid pattern (AA_reference_).

3 $${\text{PDCAAS}} = \frac{{{\text{AA}}_{\text{feedstuff}} }}{{{\text{AA}}_{\text{reference}} }} \times {\text{TD}}$$

### Plasma amino acid study with humans

Informed consent was obtained from all individual participants included in the study. The study was approved by the local ethical committee of Region Midtjylland (no. M-20110003, additional notification no. 31556).

Five male subjects (mean ± SEM; height: 185.0 ± 3.8 cm, weight: 79.2 ± 3.0 kg, age 34.6 ± 3.5 years) volunteered to participate in the study. The study was a randomized, double-blind, four-way crossover design. The four protein solutions were administered to the subjects in randomized order on four different days interspaced by at least 5 days. The four test solutions of 500 mL each contained 20 g of the respective proteins and were artificially flavored.

On trial days participants arrived at the laboratory at 8:30 AM after an overnight fast of 10 h. The subjects were allowed to drink water until 6:30 AM. During the fast the participants were instructed not to perform any strenuous activities or any kind of exercise. After reporting to the laboratory the participants rested in the supine position for 20 min while a catheter was inserted in an antecubital vein and a basal 6 mL blood sample (−5 min, pre) was collected into a Na-heparin tube. At 8:50 AM the subjects received a drink containing one of the four protein products. The drink was consumed within 2 min. The complete supplement ingestion was designated as time zero (0 min).

Blood samples were collected at time points: pre, 10, 20, 30, 45, 60, 90 and 120 min after supplement ingestion. The blood samples were all immediately centrifuged and the plasma collected and stored at −20 °C. The subjects were not allowed to eat or drink for 120 min following supplement ingestion. On one occasion a subject was, by mistake allowed to drink water following product ingestion of the Low DH supplement. This seemed to slow down the rate of plasma appearance of amino acid and this trial was excluded from all further analysis.

### Quantification of blood and plasma amino acids

Heparinized plasma samples were analysed in duplicate for amino acids by gas chromatography–mass spectrometry using the isotope dilution method (Calder et al. 1999). A working amino acid standard was prepared from a commercial amino acid mixture (AAS18; Sigma-Aldrich Denmark A/S, Brondby, Denmark) with added Gln (l-glutamine 99 %, final concentration 400 μM; Acros, Geel, Belgium). The internal standard was made from a U-13C/U-15N cell free amino acid mixture (CNLM-6696–1; Cambridge Isotope Laboratories Inc., Andover, MA). EAA analyzed were His, Ile, Leu, Lys, Met, Phe, Thr, Trp, and Val; and non-EAA analyzed were Ala, Asn, Asp, Cys, Gln, Glu, Gly, Pro, Ser, and Tyr. The method was not validated for Arg. Heparinized plasma samples were analyzed for glucose and l-lactate using d-glucose oxidase and l-lactate oxidase, respectively (YSI 7100; YSI Inc., Yellow Springs, OH). Plasma insulin was determined by time-resolved fluoroimmunometric assay (Lovendahl and Purup 2002).

### Model for protein absorption kinetics

**Fig. 2:**
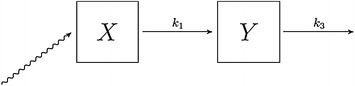
The two compartment kinetic model for the postprandial plasma concentrations of amino acid. *X* denotes the concentration of the specific amino acid or group of amino acids in the stomach while *Y* denotes the plasma concentration of the specific amino acid or group of amino acids. The rate constants *k*_1_ and *k*_3_ describes postprandial plasma appearance and clearance of the specific amino acid(s), respectively. The rate constant describing the flow of amino acids from plasma back into the stomach (*k*_2_) was assumed to be negligible

To investigate the rate of TAA, EAA and leucine plasma appearance of the four protein products, a two compartment model for the description of the postprandial plasma amino acid kinetics was applied (Fig. 2).

We first tested a model assuming first order kinetics for both the plasma appearance and clearance; however, the model systematically underestimated the peak amino acid concentrations. Instead, we found that a two compartment kinetic model, where the plasma appearance was assumed to be of zeroth order and the plasma clearance was assumed to be of first order, fitted well with the time course of the measured plasma amino acid concentrations for each subject following ingestion of each drink (see fits for TAA, EAA and leucine in the Additional file 1: Figure SIa, SIb, and SIc, respectively). We also plotted the population fitted, individual-fitted, and the individual-drink fitted concentrations of TAA, EAA and leucine estimated by the model against the actual measured amino acid concentrations (see Additional file 1: Figure SII). All these plots suggest that the chosen two compartment model provided an excellent description of the data. The compartment model with zeroth order amino acid plasma appearance also resulted in initial stomach total amino acid and leucine concentrations that were closely in line with the corresponding concentrations administered through the different drinks unlike the model with first order plasma appearance (data not shown).

The assumption of zeroth order plasma appearance means that the rate of clearance of amino acids from the stomach is described by the differential equation:

4 $$\frac{{{\text{d}}X}}{{{\text{d}}t}} = - k_{1}, \quad X_{0} = x_{0},$$

where *x*_0_ is the initial concentration of amino acids in the stomach for a specific supplement ingested by each subject, and *k*_1_ (mol L^−1^ min^−1^) is the rate constant of plasma appearance of the studied amino acids.

Similarly, the assumption of first order plasma clearance leads to the following differential equation (for description of changes in plasma amino acids levels):

5 $$\frac{{{\text{d}}Y}}{{{\text{d}}t}} = k_{1} - k_{3} \times Y_{t}, \quad Y_{0} = y_{0},$$

where *y*_0_ is the initial concentration of the AA in plasma before supplement ingestion, and *k*_3_ (min^−1^) is the rate constant of plasma clearance of AA.

Thus, the AA concentration in the stomach at time = t is given by:

6 $$X_{t} = x_{0} - k_{1} \times t, \quad t \ge 0,$$

with the restriction that the AA concentration in the stomach is zero ($X_{t} = 0$) after the stomach has been emptied at time $t = \frac{{x_{0} }}{{k_{1} }}$.

The expressions for the plasma AA concentration before and after $t = \frac{{x_{0} }}{{k_{1} }}$ are:

7 $$Y_{t} = \left\{ { \begin{array}{*{20}l} {\frac{{k_{1} }}{{k_{3} }} + \left( {y_{0} - \frac{{k_{1} }}{{k_{3} }}} \right)e^{{ - k_{3} t}} , } & {t \le \frac{{x_{0} }}{{k_{1} }}} \\ {\left[ {\frac{{k_{1} }}{{k_{3} }}e^{{\frac{{k_{3} }}{{k_{1} }}x_{0} }} + \left( {y_{0} - \frac{{k_{1} }}{{k_{3} }}} \right)} \right]e^{{ - k_{3} t}} ,} & {t > \frac{{x_{0} }}{{k_{1} }}} \\ \end{array} } \right.$$

### Statistical analysis

The subjects were not allowed to drink or eat during the postprandial measurement period but on one occasion subject no. 2 drank some water following ingestion of the Low DH supplement. Therefore all data analyses were made without subject 2, following the Low DH supplement.

The plasma amino acid data were analysed using a mixed non-linear regression model. The non-linear functional relationship between plasma AA concentration and time is presented in (7). All parameters in the non-linear expression ($$k_{1} , k_{3} , x_{0} , y_{0} )$$ were allowed to depend systematically on supplement, and random effects corresponding to subject and the interaction between subject and supplement were included for all parameters. The parameters $$k_{1} , k_{3} , x_{0} , y_{0}$$, of the model (7) were estimated by the maximum-likelihood estimation method with initial values chosen based on inspection of the individual concentration curves.

Monte Carlo simulation was used to determine 95 %-confidence intervals (CI) for AUC as well as the pairwise comparisons of the drinks with regard to AUC. More specifically this was done by simulating 50,000 curves from the joint asymptotic normal distribution of the parameter estimates, calculate the AUC for each set of parameters, and then determine the 95 %-confidence intervals from the empirical distribution of these. p values corresponding to the pairwise comparisons of the AUCs were derived under the additional assumption that parameter estimates corresponding to the different drinks were independent.

These data were analysed using R (R v 3.0.2, R core team, Vienna, Austria) with the package nlme.

Plasma insulin data were log transformed before statistical analyses to achieve normal distribution. The effects of time (pre, 10, 20, 30, 45, 60, 90, 120) and supplement (High DH, Medium DH, Low DH and Casein) and their interaction on dependent variables (glucose, and insulin levels) were assessed using a mixed-effect two-way ANOVA with repeated measurements for time and subjects (repeated measures on the same subject within supplement and time). The latter was adjusted in the model by using subject and subject × time as random effects. Linear pairwise comparisons were performed post hoc to compare differences within and between individual conditions. The level of significance was set at p < 0.05.

These data analysed using Stata (Stata v 12.1, StataCorp LP, College Station, Texas, USA) and graphs were designed in SigmaPlot (SigmaPlot v 11.0, Sysstat Software, Inc. San Jose, California, USA).

## Results

### Test protein characteristics and peptide distribution

Specifications for the four protein supplements are shown in Table 1 and chromatograms and peptide distributions for the three WPH supplements are shown in Fig. 1a–c. As shown in Fig. 1d the High DH supplement contained more free amino acids and di-and tripeptides than Medium and Low DH (free amino acids: 21 vs 2 vs 6 %, di-and tripeptides: 35 vs 11 vs 15 % in High, Medium and Low DH, respectively). The peptide distributions of the Medium and the Low DH supplements were comparable, but the Low DH supplement could contain very large peptide aggregates that were too large to pass on to the columns, and are thus not displayed on the chromatograms.

### Protein digestibility

The true digestibility was calculated using Eq. (1). The true digestibility (Table 3) was lower for the Low DH supplement (~94) than for the other three supplements (p = 0.0103). The protein digestibility corrected amino acid score (Table 3) showed that none of the EAAs were limiting the four products as compared to the FAO 2007/2011 recommendations for children 3–10 years. Thus, all PDCAAS values were above 100 and as stated by the FAO directives PDCAAS values over 100 should be considered as 100.

**Table 3:** Biological value, true digestibility, and protein digestibility corrected amino acids score (PDCAAS) for the High DH, Medium DH, Low DH, and Casein test proteins

	High DH	Medium DH	Low DH	Casein
Biological value	58 ± 2.9^a^	78 ± 1.5^b^	87 ± 0.7^c^	69 ± 1.7^d^
True digestibility	97 ± 0.5^e^	97 ± 0.4^e^	94 ± 0.7^f^	96 ± 0.4^e^
Protein digestibility corrected amino acids score*				
Histidine	140 ± 0.8	110 ± 0.4	107 ± 0.8	171 ± 0.7
Isoleucine	201 ± 1.2	181 ± 0.7	182 ± 1.3	158 ± 0.7
Leucine	264 ± 1.5	136 ± 0.5	161 ± 1.2	148 ± 0.6
Lysine	147 ± 0.8	208 ± 0.8	183 ± 1.3	161 ± 0.7
Methionine + cysteine	120 ± 0.7	175 ± 0.7	185 ± 1.3	134 ± 0.6
Phenylalanine + tyrosine	298 ± 1.7	234 ± 0.9	237 ± 1.7	216 ± 0.9
Threonine	273 ± 1.6	291 ± 1.1	267 ± 2.0	159 ± 0.7
Tryptophane	376 ± 2.1	153 ± 0.6	242 ± 1.8	173 ± 0.8
Vanline	177 ± 1.0	124 ± 0.5	130 ± 0.9	155 ± 0.7

*DH* degree of hydrolysis (% cleaved peptide bonds). *PDCAAS* protein digestibility corrected amino acids score. All values are mean ± SEM

* The FAO/WHO 2007/2011 reference pattern for 3–10 years old children was used for calculation of the PDCAAS

^a,b,c,d^Mean values within a row with unlike superscript letters were significantly different (*p* < 0.0036)

^e,f^Mean values within a row with unlike superscript letters were significantly different (*p* < 0.0298)

The biological values of the supplements differed from each other (p = 0.0044). Biological value of the Low DH supplement was highest (~87), followed by the Medium DH (~78), the Casein (~69) and the High DH (~58) supplement.

### Plasma appearance rate constants, *k*_1_, for total plasma amino acids, essential amino acids and leucine

**Fig. 3:**
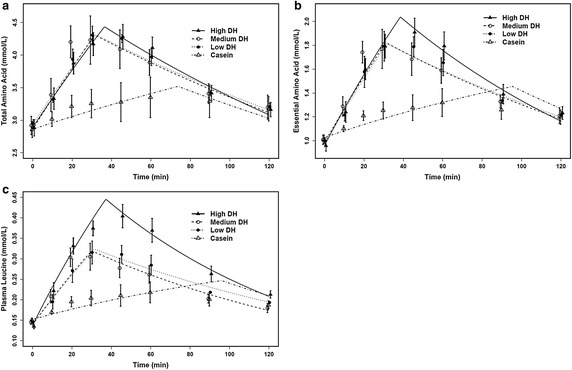
The mean curves of the kinetic model and mean concentrations for total amino acids, essential amino acids and leucine. The mean curves from the kinetic model, and the mean values ±SEM of the measured concentrations of total amino acids (**a**), essential amino acids (**b**) and leucine (**c**), are shown of each combination of protein supplement and time. DH, degree of hydrolysis (% cleaved peptide bonds). For low DH n = 4, for all other supplements n = 5

The plasma TAA, EAA and leucine concentrations for each protein product are shown in Fig. 3a, b, c respectively, along with the mean curves for each drink generated from the plasma appearance kinetic model.

The fit of the kinetic model to the measured plasma TAA, EAA and leucine concentrations for the five subjects following ingestion of the four drinks is shown in the supplemental material; figure Ia, Ib, and Ic, respectively. The mean rate constant estimates and 95 % CI of plasma appearance, *k*_1_, for TAA, EAA and leucine concentrations are shown in Table 4.

**Table 4:** Plasma appearance rate constants, *k*_1_

	High DH	Medium DH	Low DH	Casein
*k*_1_ TAA^†^	0.0585 [0.0454; 0.0754]	0.0594 [0.0459; 0.0768]	0.0560 [0.0429; 0.0732]	0.0194 [0.0129; 0.0291]*
*k*_1_ EAA^†^	0.0384 [0.0264; 0.0559]	0.0338 [0.0231; 0.0495]	0.0331 [0.0221; 0.0497]	0.0114 [0.0073; 0.0178]*
*k*_1_ Leucine^†^	0.0111 [0.0072; 0.0169]	0.0076 [0.0049; 0.0117]	0.0072 [0.0045; 0.0115]	0.0024 [0.0015; 0.0038]*

The mean rate constant estimates of plasma appearance, *k*_1_, (mol L^−1^ min^−1^) for total amino acids (TAA), essential amino acids (EAA) and leucine concentrations for the four protein supplements High DH, Medium DH, Low DH and Casein were estimated by fitting the kinetic model (Eq. 7) to the measured concentrations of TAA, EAA and leucine for each subject (low DH: n = 4, all other supplements: n = 5) following ingestion of each supplement (shown in Additional file 1: Figure SI)

* Denotes that *k*_1_ for casein is different from the three WPH supplements (p < 0.001)

^†^ *k*_1_, (mol L^−1^ min^−1^) values are means [95 %-confidence intervals]

The *k*_1_ estimates for TAA were significantly lower for casein compared to the three WPH supplements, while the *k*_1_ estimates for TAA did not differ between the three WPH products (Tables 4, 5).

**Table 5:** p values for pairwise comparisons of k_1_ for TAA, EAA and leucine for the four protein supplements

	TAA			EAA			Leucine		
	Medium DH	Low DH	Casein	Medium DH	Low DH	Casein	Medium DH	Low DH	Casein
High DH	p = 0.9008	p = 0.7183	p < 0.0001	p = 0.5532	p = 0.5120	p < 0.0001	p = 0.1516	p = 0.1266	p < 0.0001
Medium DH		p = 0.6360	p < 0.0001		p = 0.9244	p < 0.0001		p = 0.8613	p = 0.0001
Low DH			p < 0.0001			p = 0.0001			p = 0.0004

For low DH: n = 4, all other supplements: n = 5

*DH* degree of hydrolysis (% cleaved peptide bonds). *TAA* total amino acids

The estimates for the EAA plasma appearance rates, *k*_1_, were significantly lower for casein compared to the three WPH supplements, while the *k*_1_ estimates for EAA did not differ between the three WPH products (Tables 4, 5).

The estimates for the leucine plasma appearance rates, *k*_1_, were found to increase with increasing degree of hydrolysis (High DH > medium DH > low DH > Casein). However, the leucine *k*_1_—estimates were not significantly different between the three WPH supplements, while all leucine *k*_1_—estimates for the WPH products were higher than for the casein product (Tables 4, 5).

### Plasma amino acids, essential amino acids and leucine concentrations

The measured TAA mean concentrations for each protein product are shown in Fig. 3a. Overall ANOVA revealed a supplement × time-interaction (p < 0.001). At 20, 30, 45, and 60 min after supplement ingestion the TAA levels for all three WPH supplements were elevated compared to the casein supplement. No significant differences were observed between the WPH supplements.

The area under the curve (AUC) for the TAA concentration for casein was lower compared to the WPH supplements, which did not differ from each other. The TAA AUC estimates (mmol min/l) and 95 % CI were: High DH 444.95 [431.48, 477.49], Medium DH 439.51 [406.89, 473.04], Low DH 442.66 [406.81, 480.06], Casein 386.86 [357.52, 418.11].

The plasma EAA mean concentrations, for each supplement, are shown in Fig. 3b. Overall ANOVA revealed a supplement × time-interaction (p < 0.001). At 45, and 60 min the EAA level for the high DH was greater than for medium DH (p < 0.05). Moreover, at 20, 30, 45, and 60 min after supplement ingestion the EAA levels for all three WPH supplements were elevated compared to the casein supplement.

The AUC for the EAA concentration for casein was lower compared to the WPH supplements, which did not differ from each other. The EAA AUC estimates (mmol min/l) and 95 % CI were: High DH 185.25 [171.45, 200.62], Medium DH 175.31 [162.88, 189.17], Low DH 175.47 [162.24, 190.27], Casein 152.84 [139.26, 165.81].

The plasma leucine mean concentrations for each protein product are shown in Fig. 3c. Overall ANOVA revealed a supplement × time-interaction (p < 0.001). Differences between the three WPH supplements were observed, at 30, 45, and 60 min, in which the leucine level for high DH was greater than for both medium and low. With regards to casein, at 10 min following supplement ingestion the leucine level for High DH was elevated compared to the casein supplement. Moreover, at 20, 30, 45, and 60 min after supplement ingestion the leucine levels for all three WPH supplements were elevated compared to the casein supplement. At 90 min the leucine level for High DH remained elevated compared to the casein supplement, while at 120 min there were no differences in the plasma leucine levels between the four products.

The AUC for the leucine concentration in the casein supplement was significantly lower than for the three WPH supplements. Moreover the leucine AUC from the high DH supplement was greater than the medium, low DH, and casein supplements. Leucine AUC estimates (mmol min/l) and 95 % CI: High DH 36.83 [33.67, 40.21], Medium DH 28.43 [26.37, 30.71], Low DH 29.70 [27.26, 32.44], Casein 24.93 [21.58, 27.60].

### Plasma glucose and insulin responses

**Fig. 4:**
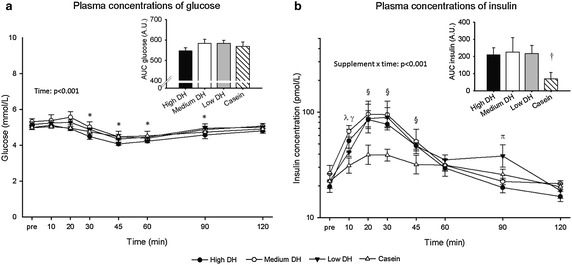
Plasma concentrations of glucose and insulin. DH, degree of hydrolysis (% cleaved peptide bonds). AUC, Area under the curve. AU, Arbitrary units. **a** The plasma glucose concentrations are presented as mean ± SEM for the three whey protein hydrolysates (WPH) supplements (High DH, Medium DH, Low DH) and a casein supplement at pre, and 10, 20, 30, 45, 60, 90, and 120 min after supplement ingestion. An overall effect of time was found (p < 0.001). *Denotes that concentrations of the four protein sources were lower than the pre level. The *insert figure* shows area under the curve for plasma glucose concentrations of the four protein supplements. For low DH n = 4, for all other supplements n = 5. **b** The plasma insulin concentrations are presented on a log scale as geometric means ± back-transformed ± SEM for the three WPH supplements (High DH, Medium DH, Low DH) and a casein supplement at pre, and 10, 20, 30, 45, 60, 90, and 120 min after supplement ingestion. An interaction was found for supplement × time (p < 0.001). ^*λ*^Denotes that High DH and Medium DH were higher than Casein (p < 0.003). ^*γ*^Denotes that Medium DH was higher than Low DH (p = 0.016). ^§^Denotes that the three WPH supplements were higher than the casein supplement (p < 0.023). ^π^Denotes that Low DH was higher than all other supplements (p < 0.028). The *insert figure* shows area under the curve for plasma insulin concentrations of the four protein supplements. ^†^Denotes that Casein was lower than all three WPH supplements. For low DH n = 4, for all other supplements n = 5

For the plasma glucose concentrations a significant main effect of time (p < 0.001) was observed, with plasma glucose concentrations responding similarly (p = 0.846) for all four plements (Fig. 4a).

The AUC for the insulin concentration of casein was significantly lower than for the three WPH supplements (p < 0.05), which did not differ from each other (insert figure in Fig. 4b). Insulin AUC estimates and 95 % CI were: High DH 210.6 [133.3, 287.9], Medium DH 226.0 [61.6, 390.4], Low DH 217.1 [124.1, 310.1], Casein 69.2 [-6.2, 144.7].

## Discussion

In the present study we investigated if the degree of whey protein hydrolysis affects the postprandial rate of plasma amino acids appearance. Three whey protein hydrolysates with varying degrees of hydrolysis were compared and a casein protein was included as reference. We found that the degree of hydrolysis does not seem to constitute a pivotal factor for the postprandial rate of amino acid appearance of whey protein hydrolysates within the studied range of hydrolysis.

### Digestibility

Before comparing the absorption kinetics of the four protein fractions, the nutritional quality of the protein fractions was assessed in a rat study. The true digestibility (i.e. the amount of protein absorbed from the nourishment), was found to be high (>94) for all proteins fractions. Furthermore the content of each of the EAAs in all four protein products more than fulfilled the requirement pattern, i.e., the PDCAAS score was above 100 for all proteins products. This indicates that all four protein fractions were digestible and fully met the human requirement for EAAs.

The biological value of the proteins was measured to assess bioavailability of the proteins (i.e. the amount of protein that was retained in the rats, and thus not excreted in urine and faeces). The true digestibility only shows if the protein can be absorbed from the intestines and not if it is retained and utilized in the rat. Dairy protein processing procedures such as heat and alkaline treatments of the protein have been reported to produce compounds such as d-amino acids and lysinoalanine that have a considerably impaired digestibility (Desrosiers and Savoie 1991; Friedman 1999). Racemization of amino acids may not affect the absorption of a given protein, but the bioavailability of the protein may be considerably impaired (Sarwar Gilani et al. 2012). The score provided by the PDCAAS method has been criticized for not accounting for the bioavailability of proteins (Sarwar Gilani et al. 2012). Furthermore, it can be questioned if the protein bioavailability assessed in rats can be compared to bioavailability in humans. Rats have a higher need for the sulphur containing amino acid methionine and cysteine than humans to maintain the condition of the fur. The four protein products contained more EAAs than required by the rats, except for the methionine and cysteine amino acids. Growing rats need 6.5 g of methionine + cysteine/100 g total protein (Council 1995), however, the high, medium, low DH and casein supplements contained 2.8, 3.9, 4.3, and 3.2 g of methionine + cysteine/100 g of total protein. In the present study, the biological value of the protein fractions was found to decrease considerably with increasing degree of hydrolysis and decreasing content of methionine + cysteine/g total protein. Therefore, the lower biological value associated with higher DH (High DH ~33 % lower than Low DH and Medium DH ~10 % lower than Low DH) presumably relates to the insufficient amounts of methionine and cysteine that did not meet the requirements of rats. Since rats have higher requirements for methionine and cysteine than humans the biological value assessed in rats may not be completely transferable to humans. Moreover, all four protein supplements more than fulfilled the EAA requirements for EAA of pre-school children of all EAA.

### Amino acid plasma appearance

To assess whether different degrees of hydrolysis would differentially affect the rate of plasma appearance, we applied a two compartment model for the description of the postprandial plasma amino acid appearance. Importantly, whole body protein kinetics are complex and involves several protein pools that are dynamically interrelated, and not only a postprandial supply of amino acid affects the protein pool in the plasma (Stoll and Burrin 2006). Furthermore, the tissue of the splanchnic bed, which comprises the liver and the portal-drained viscera, has been shown to extract nearly 60 % of dietary nitrogen (Fouillet et al. 2003) and the splanchnic bed may extract different amounts of the individual amino acids (Stoll and Burrin 2006). Thus, investigation of the metabolic fate of ingested amino acids would necessitate tracer methodologies with two different tracers (Beaufrere et al. 1992; Stoll and Burrin 2006). The two compartment kinetic model applied in this study was designed to quantify the rate of amino acid increases in the plasma pool, disregarding the actual origin of the amino acids. We focused on the rate of amino acid appearance, since rapid aminoacidemia has been suggested to be important for maximally stimulating MPS (Phillips 2014). The model was found to fit well with the measurements of total amino acids for each individual (Additional file 1: Figure SIa). Furthermore, the plots of the population fitted, individual-fitted, and the individual-drink fitted concentrations estimated by the kinetic model against the actual measured concentrations of total plasma amino acids (Additional file 1: Figure SII), confirmed that the model described the concentration–time course for each supplement well. Plasma appearance rate constants, *k*_1_, were estimated from the model for each supplement. The three WPH products had a relatively high degree of hydrolysis (DH range: 23–48 %), however, the High DH supplement was especially rich in di- and tri-peptides (35 %, 175–375 kDa) as compared to the Medium and Low DH supplements (11 and 15 %, respectively). Studies in human subjects, which comprise perfusion of a segment of the small intestine have shown, that dipeptides are usually absorbed faster than a corresponding mixture of the same amino acids provided in free form (Adibi 1971). This may be attributed to the very high capacity of the highly specific di- and tripeptide transporter PEPT1 that resides in the apical membrane of enterocytes (Daniel 2004). We expected that the high content of di- and tripeptides in the High DH supplement would favour a faster rate of plasma appearance than for the Medium and Low DH proteins. Consequently, it was expected that the plasma appearance rate constant, *k*_1_, for High DH would be greater than from the two other WPH supplements. However, only small differences were observed between WPH supplements. Although immediately surprising, this may be explained by too small a relative difference in di- and tripeptide content between the WPH supplement to evoke substantial differences in the plasma appearance rate constants. Another explanation may relate to the endogenous enzymatic hydrolysis in the gut, which may overrule the initial differences of the degree of hydrolysis to produce similar absorption rates. Moreover, it should be recognized that the absorption process is complex since some amino acids are metabolized by intestinal cells whereas others are metabolized in the liver, and furthermore the individual amino acids are absorbed at divergent rates (Bertolo and Burrin 2008; Stoll and Burrin 2006). Finally, we must acknowledge that we did not include an intact whey protein reference and therefore we cannot conclude on the effects of protein hydrolysis on plasma appearance rates per se.

In relation to casein, the three WPHs had more than 2.9-fold higher TAA plasma appearance rates than the casein reference protein, which lead to a ~2.3-fold higher AUC for the WPH compared to casein. These observations are in accordance with previous studies reporting that whey protein promote fast and high increases of plasma amino acid concentrations compared to casein during the first hour following ingestion (Burd et al. 2012; Reitelseder et al. 2011).

An important aspect relates to the traditional distinction between essential and non-essential amino acids, a distinction that has recently been challenged. We whish to emphasize that in the present paper we have primarily focused on digestion and absorption in relation to TAAs and EAAs. In the current context, we feel that this distinction is justified because of earlier studies demonstrating that these specific EAAs are able to drive the activation of MPS and to accentuate exercise-induced activation of MPS in healthy human adult skeletal muscle (Borsheim et al. 2002; Tipton et al. 1999a). However, we also want to stress that the traditionally termed non-EAAs (e.g. glutamine and arginine), are recently contended to possess important and essential functions in many other tissues and conditions, such as immune metabolism and blood-flow regulation (Hou et al. 2015; Wu et al. 2013). Thus, while we have not provided explicit data on the non-EAAs plasma appearance in the present paper, these amino acids may still hold important functions and their roles in should be further investigated.

As for the rat as well as the human data, it should be noted that the number of animals/subjects was relatively low, which of course increase the chance of the type 2 error. On the other hand, the samples sizes employed for both animal/subject experiments are similar to those employed in previous similar studies (Biolo et al. 1997; Morifuji et al. 2010).

## Conclusion

Within the studied range of hydrolysis we were unable to demonstrate that the degree of hydrolysis constitutes a pivotal factor for the postprandial rate of amino acid appearance of whey protein hydrolysates.

## Additional file

10.1186/s40064-016-1995-x The kinetic model (*solid line*) that was described by Eq. 7 was fitted to the measured plasma **SIa** total amino acids (TAA), **SIb** essential amino acids (EAA), and **SIc** leucine concentrations (*open circles*) for each subject 1–5 following ingestion of the supplements: High DH, Medium DH, Low DH and Casein. Time points for measurements were: prior to and at 10, 20, 30, 45, 60, 90, and 120 min after ingestion of the supplement. From these fits of the mean estimated rate constant of plasma appearance, *k*_1_, of TAA, EAA and leucine for each supplement was found. Measurements for the Low DH supplement for subject 2 were excluded from the *k*_1_ estimation since the subject by a mistake ingested water following supplement ingestion. This may have delayed the plasma appearance of TAA, EAA, and leucine. **Figure SIa.** The fit of the kinetic model to the total amino acid concentration for each supplement and subject. **Figure SIb.** The fit of the kinetic model to the essential amino acid concentration for each supplement and subject. **Figure SIc.** The fit of the kinetic model to the leucine concentration for each supplement and subject. **Figure SII.** Population fitted, individual-fitted, and the individual-drink fitted concentrations estimated by the kinetic model are plottet against the actual measured concentrations of total amino acids (*upper panel*), essential amino acids (*middle panel*), and leucine (*lower panel*).

## References

[CR1] Adibi SA (1971). Intestinal transport of dipeptides in man: relative importance of hydrolysis and intact absorption. J Clin Invest.

[CR2] Beaufrere B, Fournier V, Salle B, Putet G (1992). Leucine kinetics in fed low-birth-weight infants—importance of splanchnic tissues. Am J Physiol.

[CR3] Bertelsen H, Langborg WP (2012) Galacto-oligosaccharide-containing composition and a method of producing it. Google Patents

[CR4] Bertolo RF, Burrin DG (2008). Comparative aspects of tissue glutamine and proline metabolism. J Nutr.

[CR5] Biolo G, Tipton KD, Klein S, Wolfe RR (1997). An abundant supply of amino acids enhances the metabolic effect of exercise on muscle protein. Am J Physiol.

[CR6] Boirie Y, Dangin M, Gachon P, Vasson MP, Maubois JL, Beaufrere B (1997). Slow and fast dietary proteins differently modulate postprandial protein accretion. Proc Natl Acad Sci USA.

[CR7] Borsheim E, Tipton KD, Wolf SE, Wolfe RR (2002). Essential amino acids and muscle protein recovery from resistance exercise. Am J Physiol Endocrinol Metab.

[CR8] Burd NA, Yang Y, Moore DR, Tang JE, Tarnopolsky MA, Phillips SM (2012). Greater stimulation of myofibrillar protein synthesis with ingestion of whey protein isolate vs. micellar casein at rest and after resistance exercise in elderly men. Br J Nutr.

[CR9] Calder AG, Garden KE, Anderson SE, Lobley GE (1999). Quantitation of blood and plasma amino acids using isotope dilution electron impact gas chromatography/mass spectrometry with U-(13)C amino acids as internal standards. Rapid Commun Mass Spectrom RCM.

[CR10] Churchward-Venne TA (2012). Supplementation of a suboptimal protein dose with leucine or essential amino acids: effects on myofibrillar protein synthesis at rest and following resistance exercise in men. J Physiol.

[CR11] Churchward-Venne TA (2014). Leucine supplementation of a low-protein mixed macronutrient beverage enhances myofibrillar protein synthesis in young men: a double-blind, randomized trial. Am J Clin Nutr.

[CR12] Council NR (1995). Nutrient Requirements of the laboratory rat.

[CR13] Crozier SJ, Kimball SR, Emmert SW, Anthony JC, Jefferson LS (2005). Oral leucine administration stimulates protein synthesis in rat skeletal muscle. J Nutr.

[CR14] Cuthbertson D (2005). Anabolic signaling deficits underlie amino acid resistance of wasting, aging muscle. FASEB J.

[CR15] Daniel H (2004). Molecular and integrative physiology of intestinal peptide transport. Annu Rev Physiol.

[CR16] Dennis MD, Baum JI, Kimball SR, Jefferson LS (2011). Mechanisms involved in the coordinate regulation of mTORC1 by insulin and amino acids. J Biol Chem.

[CR17] Desrosiers T, Savoie L (1991). Extent of damage to amino acid availability of whey protein heated with sugar. J Dairy Res.

[CR18] Eggum BO (1973). A study of certain factors influencing protein utilisation in rats and pigs.

[CR19] Fouillet H, Gaudichon C, Bos C, Mariotti F, Tome D (2003). Contribution of plasma proteins to splanchnic and total anabolic utilization of dietary nitrogen in humans. Am J Physiol Endocrinol Metab.

[CR20] Friedman M (1999). Chemistry, nutrition, and microbiology of d-amino acids. J Agric Food Chem.

[CR21] Hansen B (1989). Determination of nitrogen as elementary-n, an alternative to Kjeldahl. Acta Agr Scand.

[CR22] Hou Y, Yin Y, Wu G (2015). Dietary essentiality of “nutritionally non-essential amino acids” for animals and humans. Exp Biol Med (Maywood).

[CR23] International Dairy Federation—Milk Determination of Fat-Content—Gravimetric Method (Reference Method)—Provisional International Idf Standard Ib 1983 (1983) Milchwissenschaft 38:720–725

[CR24] Jørgensen H, Gabert VM, Eggum O (1997). The nutritional value of high-lysine barley determined in rats, young pigs and growing pigs. J Sci Food Agric.

[CR25] Lovendahl P, Purup HM (2002). Technical note: time-resolved fluoro-immunometric assay for intact insulin in livestock species. J Anim Sci.

[CR26] Moberg M, Apro W, Ohlsson I, Ponten M, Villanueva A, Ekblom B, Blomstrand E (2014). Absence of leucine in an essential amino acid supplement reduces activation of mTORC1 signalling following resistance exercise in young females. Appl Physiol Nutr Metab.

[CR27] Morifuji M (2010). Comparison of different sources and degrees of hydrolysis of dietary protein: effect on plasma amino acids, dipeptides, and insulin responses in human subjects. J Agric Food Chem.

[CR28] Nielsen PM, Petersen D, Dambmann C (2001). Improved method for determining food protein degree of hydrolysis. J Food Sci.

[CR29] Phillips SM (2014). A brief review of critical processes in exercise-induced muscular hypertrophy sports medicine.

[CR30] Power O, Hallihan A, Jakeman P (2009). Human insulinotropic response to oral ingestion of native and hydrolysed whey protein. Amino Acids.

[CR31] Proud CG (2014). Control of the translational machinery by amino acids. Am J Clin Nutr.

[CR32] Regulation TCOTEC-C (2009). Commission Regulation (EC) No 152/2009.

[CR33] Reitelseder S (2011). Whey and casein labeled with l-[1-13C]leucine and muscle protein synthesis: effect of resistance exercise and protein ingestion American journal of physiology. Endocrinol Metab.

[CR34] Rutherfurd SM, Moughan PJ (2012). Available versus digestible dietary amino acids. Br J Nutr.

[CR35] Sarwar Gilani G, Wu Xiao C, Cockell KA (2012). Impact of antinutritional factors in food proteins on the digestibility of protein and the bioavailability of amino acids and on protein quality. Br J Nutr.

[CR36] Schaafsma G (2005). The protein digestibility-corrected amino acid score (PDCAAS)—a concept for describing protein quality in foods and food ingredients: a critical review. J AOAC Int.

[CR37] Stoll B, Burrin DG (2006). Measuring splanchnic amino acid metabolism in vivo using stable isotopic tracers. J Anim Sci.

[CR38] Suryawan A, Jeyapalan AS, Orellana RA, Wilson FA, Nguyen HV, Davis TA (2008). Leucine stimulates protein synthesis in skeletal muscle of neonatal pigs by enhancing mTORC1 activation American journal of physiology. Endocrinol Metab.

[CR39] Tang JE, Moore DR, Kujbida GW, Tarnopolsky MA, Phillips SM (2009). Ingestion of whey hydrolysate, casein, or soy protein isolate: effects on mixed muscle protein synthesis at rest and following resistance exercise in young men. J Appl Physiol (Bethesda, Md: 1985).

[CR40] Tipton KD, Ferrando AA, Phillips SM, Doyle D, Wolfe RR (1999). Postexercise net protein synthesis in human muscle from orally administered amino acids. Am J Physiol.

[CR41] Tipton KD, Gurkin BE, Matin S, Wolfe RR (1999). Nonessential amino acids are not necessary to stimulate net muscle protein synthesis in healthy volunteers. J Nutr Biochem.

[CR42] Volpi E, Kobayashi H, Sheffield-Moore M, Mittendorfer B, Wolfe RR (2003). Essential amino acids are primarily responsible for the amino acid stimulation of muscle protein anabolism in healthy elderly adults. Am J Clin Nutr.

[CR43] West DW (2011). Rapid aminoacidemia enhances myofibrillar protein synthesis and anabolic intramuscular signaling responses after resistance exercise. Am J Clin Nutr.

[CR44] Wu G (2013). Dietary requirements of “nutritionally non-essential amino acids” by animals and humans. Amino Acids.

