# Supplementary material for: Effect of degree of hydrolysis of whey protein on in vivo plasma amino acid appearance in humans
Source: Springerplus. 2016 Mar 31;5:382. doi: 10.1186/s40064-016-1995-x (PMC4814394; doi:10.1186/s40064-016-1995-x)

Additional file 1

Fig SIa-c. The kinetic model (solid line) that was described by equation 7 was fitted to the measured plasma (**SIa**) total amino acids (TAA), (**SIb**) essential amino acids (EAA), and (**SIc**) leucine concentrations (open circles) for each subject 1-5 following ingestion of the supplements: High DH, Medium DH, Low DH and Casein. Time points for measurements were: prior to and at 10, 20, 30, 45, 60, 90, and 120 min after ingestion of the supplement. From these fits of the mean estimated rate constant of plasma appearance, k_1, of TAA, EAA and leucine for each supplement was found.

NB. Measurements for the Low DH supplement for subject 2 were excluded from the k1 estimation since the subject by a mistake ingested water following supplement ingestion. This may have delayed the plasma appearance of TAA, EAA, and leucine.

Fig SIa The fit of the kinetic model to the total amino acid concentration for each supplement and subject

Fig SIb The fit of the kinetic model to the essential amino acid concentration for each supplement and subject

Fig SIc The fit of the kinetic model to the leucine concentration for each supplement and subject

Fig SIa

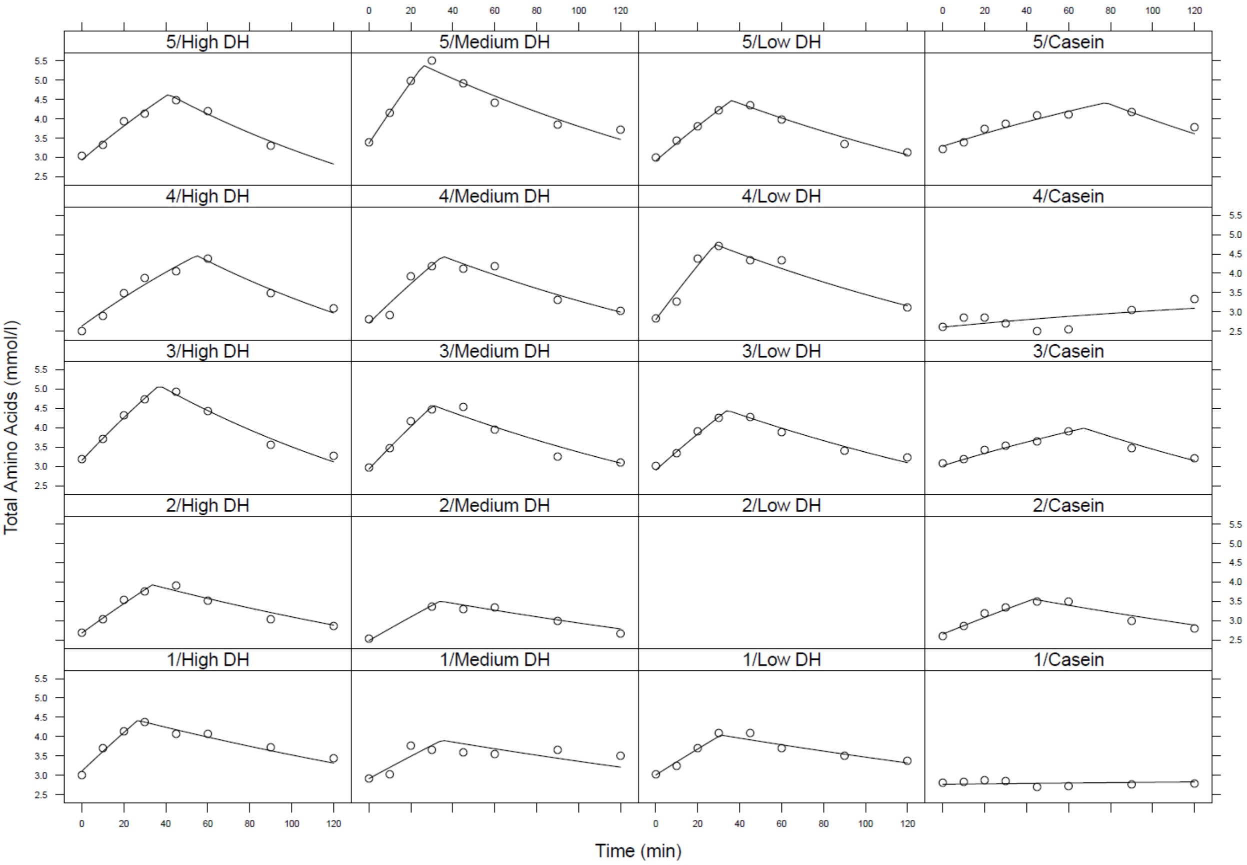

5/High DH
5/Medium DH
5/Low DH
5/Casein
4/High DH
4/Medium DH
4/Low DH
4/Casein
3/High DH
3/Medium DH
3/Low DH
3/Casein
2/High DH
2/Medium DH
2/Low DH
2/Casein
1/High DH
1/Medium DH
1/Low DH
1/Casein
Total Amino Acids (mmol/l)
Time (min)

Fig SIb

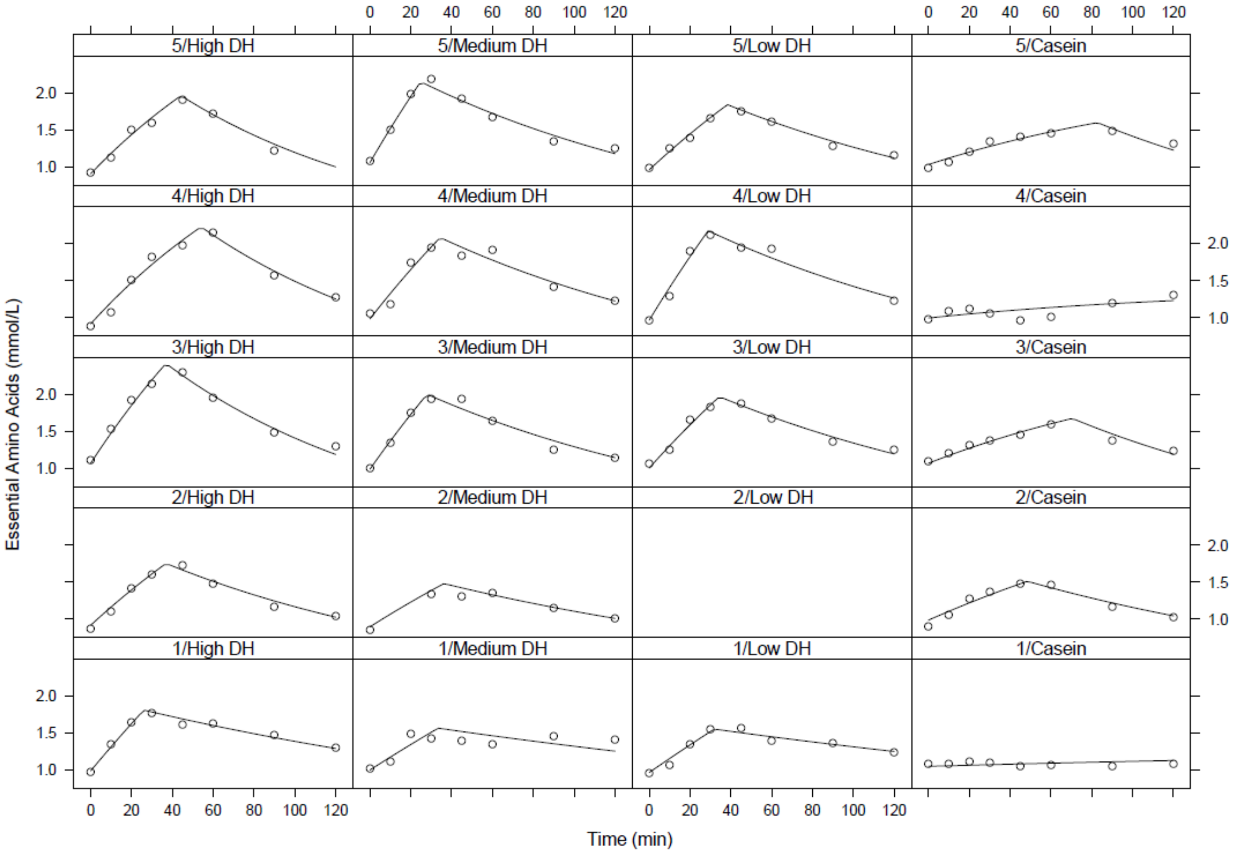
5/High DH
5/Medium DH
5/Low DH
5/Casein
4/High DH
4/Medium DH
4/Low DH
4/Casein
3/High DH
3/Medium DH
3/Low DH
3/Casein
2/High DH
2/Medium DH
2/Low DH
2/Casein
1/High DH
1/Medium DH
1/Low DH
1/Casein
Essential Amino Acids (mmol/L)
Time (min)
0 20 40 60 80 100 120
1.0 1.5 2.0

Fig SIc

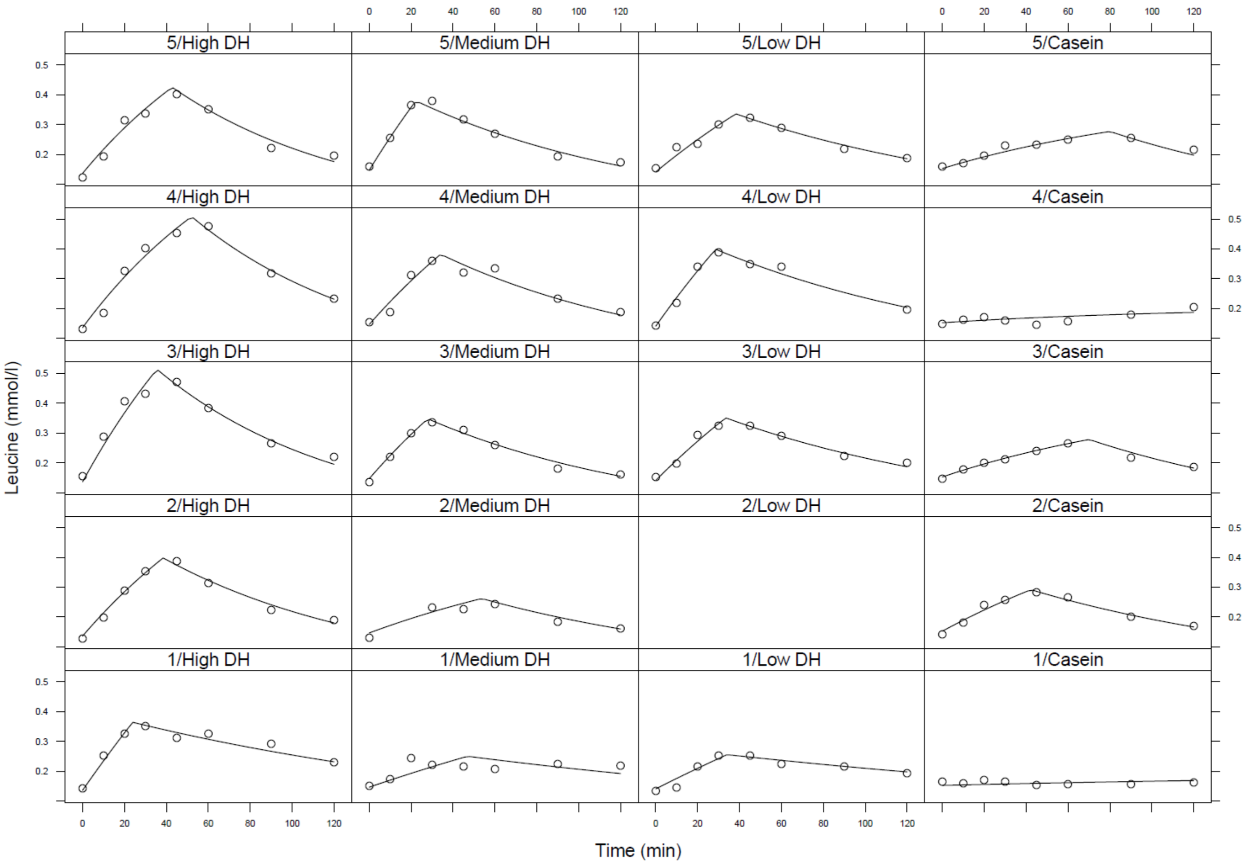
5/High DH
5/Medium DH
5/Low DH
5/Casein
4/High DH
4/Medium DH
4/Low DH
4/Casein
3/High DH
3/Medium DH
3/Low DH
3/Casein
2/High DH
2/Medium DH
2/Low DH
2/Casein
1/High DH
1/Medium DH
1/Low DH
1/Casein
Leucine (mmol/l)
Time (min)
0 20 40 60 80 100 120
0.2 0.3 0.4 0.5

Fig SII

Population fitted, individual-fitted, and the individual-drink fitted concentrations estimated by the kinetic model are plottet against the actual measured concentrations of total amino acids (upper panel), essential amino acids (middle panel), and leucine (lower panel).

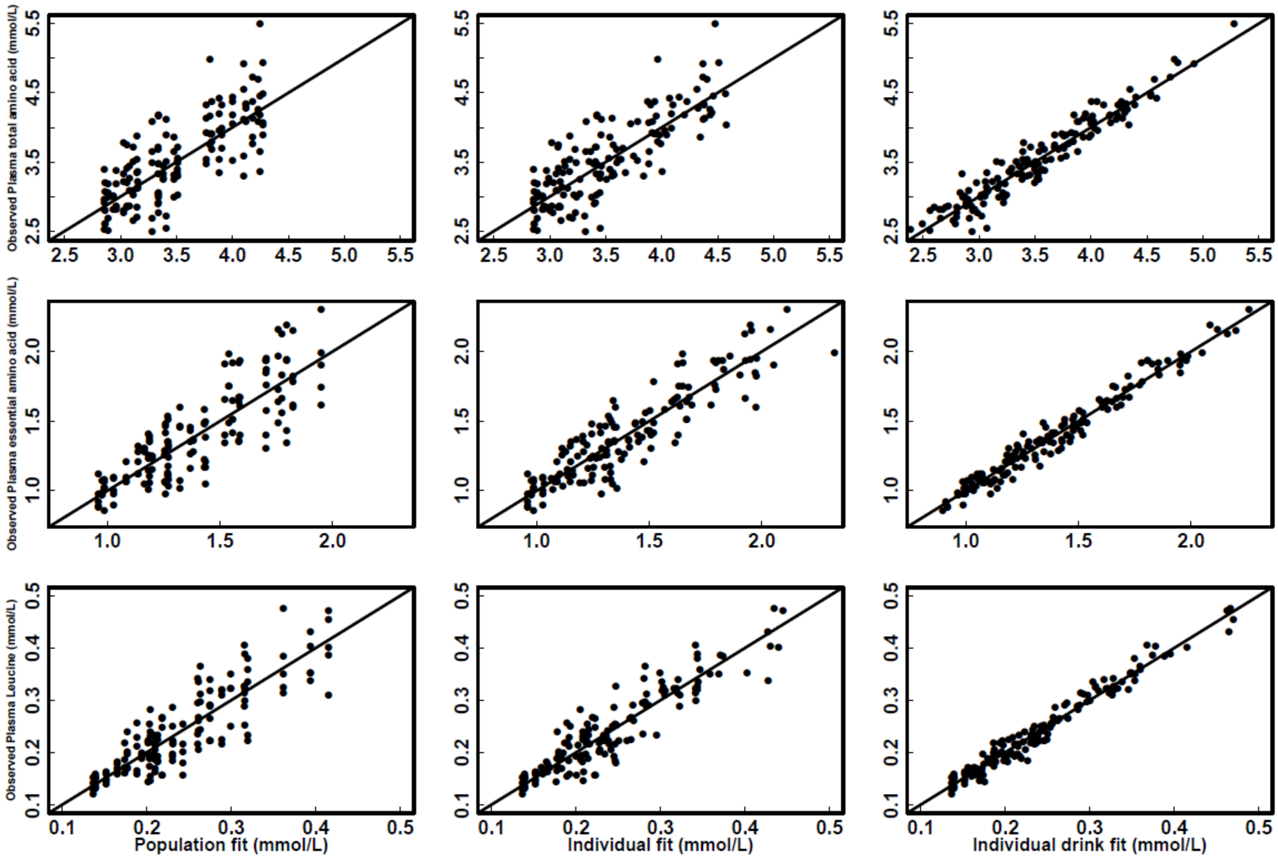

Supplement: Supplementary file 1 — 10.1186/s40064-016-1995-x The kinetic model (solid line) that was described by Eq. 7 was fitted to the measured plasma SIa total amino acids (TAA), SIb essential amino acids (EAA), and SIc leucine concentrations (open circles) for each subject 1–5 following ingestion of the supplements: High DH, Medium DH, Low DH and Casein. Time points for measurements were: prior to and at 10, 20, 30, 45, 60, 90, and 120 min after ingestion of the supplement. From these fits of the mean estimated rate constant of plasma appearance, k 1, of TAA, EAA and leucine for each supplement was found. Measurements for the Low DH supplement for subject 2 were excluded from the k 1 estimation since the subject by a mistake ingested water following supplement ingestion. This may have delayed the plasma appearance of TAA, EAA, and leucine. Figure SIa. The fit of the kinetic model to the total amino acid concentration for each supplement and subject. Figure SIb. The fit of the kinetic model to the essential amino acid concentration for each supplement and subject. Figure SIc. The fit of the kinetic model to the leucine concentration for each supplement and subject. Figure SII. Population fitted, individual-fitted, and the individual-drink fitted concentrations estimated by the kinetic model are plottet against the actual measured concentrations of total amino acids (upper panel), essential amino acids (middle panel), and leucine (lower panel). [file 40064_2016_1995_MOESM1_ESM.pdf]
